# Insights gained from gene therapy in animal models of retGC1 deficiency

**DOI:** 10.3389/fnmol.2014.00043

**Published:** 2014-05-14

**Authors:** Shannon E. Boye

**Affiliations:** Department of Ophthalmology, University of FloridaGainesville, FL, USA

**Keywords:** LCA1, Leber congenital amaurosis, *GUCY2D*, retGC1, GC1, guanylate cyclase, retinal gene therapy, AAV

## Abstract

Vertebrate species possess two retinal guanylate cyclases (retGC1 and retGC2) and at least two guanylate cyclase activating proteins (GCAPs), GCAP1 and GCAP2. GCAPs function as Ca^2+^ sensors that regulate the activity of guanylate cyclases. Together, these proteins regulate cGMP and Ca^2+^ levels within the outer segments of rod and cone photoreceptors. Mutations in *GUCY2D*, the gene that encodes retGC1, are a leading cause of the most severe form of early onset retinal dystrophy, Leber congenital amaurosis (LCA1). These mutations, which reduce or abolish the ability of retGC1 to replenish cGMP in photoreceptors, are thought to lead to the biochemical equivalent of chronic light exposure in these cells. In spite of this, the majority of LCA1 patients retain normal photoreceptor laminar architecture aside from foveal cone outer segment abnormalities, suggesting they may be good candidates for gene replacement therapy. Work began in the 1980s to characterize multiple animal models of retGC1 deficiency. 34 years later, all models have been used in proof of concept gene replacement studies toward the goal of developing a therapy to treat *GUCY2D*-LCA1. Here we use the results of these studies as well as those of recent clinical studies to address specific questions relating to clinical application of a gene therapy for treatment of LCA1.

## INTRODUCTION

**FIGURE 1:**
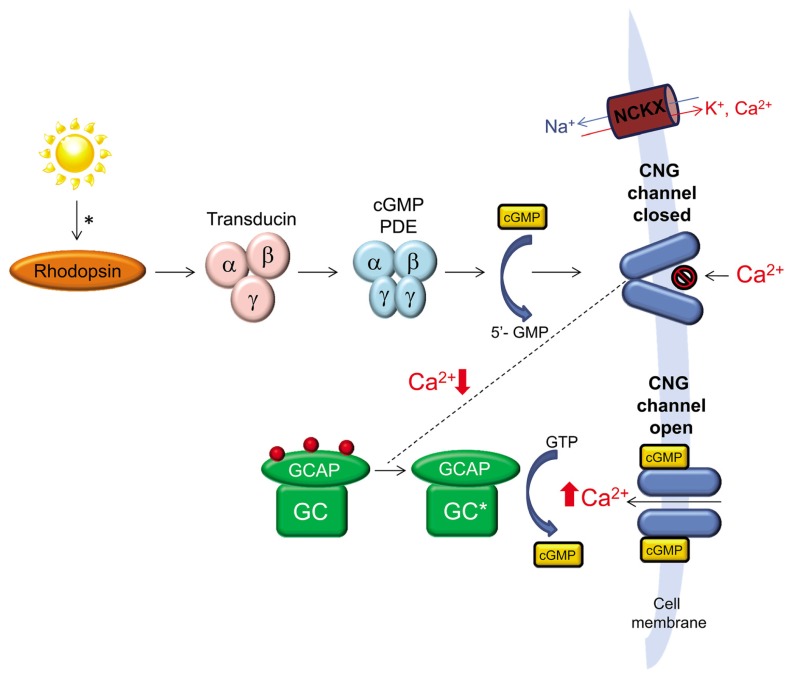
**The role of retinal guanylate cyclase (retGC) in phototransduction.** A photon of light activates rhodopsin initiating a cascade of events, the end result of which is reduction of cGMP, closure of cGMP-gated channels and reduction of intracellular Ca^2+^ in photoreceptor outer segments. Continued expulsion of Ca^2+^ by Na^+^/Ca^2+^-K^+^ exchangers (NCKX) results in activation of retGC by GCAP. retGC replenishes intracellular cGMP which reopens cGMP-gated channels allowing for Ca^2+^ influx and a return of the photoreceptor to its dark-adapted state.

The ability to process light into an electrochemical signal depends on the precise regulation of levels of cGMP and Ca^2+^ within the outer segments of rod and cone photoreceptor cells. In the absence of light stimulation, intracellular cGMP and Ca^2+^ levels are high and a continuous flow of Na^+^ and Ca^2+^ ions through cGMP-gated channels and Na^+^/Ca^2+^ exchangers keeps the cell in a depolarized state. Activation of rhodopsin or cone opsins in rod and cone outer segments, respectively, leads to activation of transducin that in turn activates cGMP phosphodiesterase (PDE) leading to a reduction in the concentration of cGMP in the outer segment. Reduction of cGMP leads to closure of cGMP-gated channels, reduced Na^+^/Ca^2+^ influx and hyperpolarization of the cell (Pugh et al., 1997). Recovery of light stimulated photoreceptors to the dark state is hastened by the decrease in intracellular Ca^2+^ that results from continued expulsion of Ca^2+^ by Na^+^/Ca^2+^-K^+^ exchangers. Calcium has several roles in photoreceptor cells, one of which is to regulate the activity of retinal guanylate cyclases, retGC1 and retGC2 (Koch and Stryer, 1988). It does so by binding to guanylate cyclase activating proteins (GCAPs), a family of calcium-binding proteins that regulate the activity of retGCs (Polans et al., 1996). Two variants of guanylate cyclases (retGC1 and retGC2) and at least two variants of GCAPs (GCAP1 and GCAP2) are present in the outer segments of vertebrate photoreceptors (Shyjan et al., 1992; Margulis et al., 1993; Dizhoor et al., 1994; Goraczniak et al., 1994, 1997; Palczewski et al., 1994; Lowe et al., 1995; Yang et al., 1995; Imanishi et al., 2004). In the dark, high levels of intracellular Ca^2+^ promote its binding to GCAP and inhibit the ability of this protein to activate retGCs. Reduction of free, intracellular Ca^2+^ following light stimulation leads to a decrease in the amount of Ca^2+^ bound to GCAP in exchange for Mg^2+^, which allows this protein to activate retGCs (Peshenko and Dizhoor, 2006; **Figure 1**). *In vivo*, retGC1 is the preferred target of GCAP1 (Olshevskaya et al., 2012). *In vitro*, some studies show that GCAP1 and GCAP2 can activate both retGCs, albeit with slightly different affinities and sensitivity to Ca^2+^ (Hwang et al., 2003; Peshenko and Dizhoor, 2004; Peshenko et al., 2011) while another shows that GCAP1 cannot activate retGC2 (Haeseleer et al., 1999).

Mutations in *GUCY2D*, the gene that encodes retGC1, are associated with the severe, early onset, autosomal recessive disorder Leber congenital amaurosis-1 (LCA1; Perrault et al., 1996, 2000). LCA1 causing mutations are found throughout *GUCY2D*, can alter the enzyme’s structure or stability, affect transport of other peripheral membrane associated protein, and are frequently null (Karan et al., 2010; Jacobson et al., 2013). Without fully functional retGC1 to replenish intracellular cGMP, as is the case in LCA1, cGMP-gated cation channels will remain closed as they are in the light stimulated state. Hence, mutations in *GUCY2D* are thought to induce the biochemical equivalent of chronic light exposure in photoreceptors. LCA1 patients routinely present with severely reduced visual acuities, attenuated or extinguished electroretinogram (ERG), nystagmus, digito-ocular signs, and apparently normal fundus appearance (Perrault et al., 1999a, 2000; Hanein et al., 2004; Yzer et al., 2006; den Hollander et al., 2008). While early reports indicated retinal degeneration was associated with this form of LCA (Milam et al., 2003; Porto et al., 2003), later studies revealed preservation of retinal laminar architecture in LCA1 patients (Simonelli et al., 2007; Pasadhika et al., 2010; Jacobson et al., 2013).

Preservation of retinal structure despite profound visual disturbance suggests that LCA1 patients would be good candidates for gene replacement therapy. Before initiating such clinical trials there are major questions that need to be answered. Here we stepwise address these questions by reviewing findings of proof of concept studies utilizing different animal models of retGC1 deficiency (results summarized in **Table 1**). Additionally, we provide an update on efforts to clinically apply a gene therapy for LCA1.

**Table 1:** Summary of proof of concept experiments.

Animal model	Vector	Treatment age	ERG results	Behavior outcomes	Structural results	Length of study
GUCY1*B chicken	LV-EF1α-*bGC1*	E2	Cones/rods: up to 6% of WT	Cone-mediated OKN reflex/volitional behavior	Degeneration slowed	5 weeks
GC1KO mouse	AAV5-smCBA-*Gucy2e*	P14–P25	Cones: up to ~45% of WT	Cone-mediated OKN reflex	Cone preservation	3 months, 1 year
	AAV5-hGRK1-*Gucy2e*	P14–P25	Cones: up to ~45% of WT	Cone-mediated OKN reflex	Cone preservation	3 months, 1 year
	AAV8-hGRK1-*GUCY2D*	P10	Cones: up to ~65% of WT	Cone-mediated OKN reflex	Cone preservation	6 months
			Rods: up to 100% of WT			
	AAV8(Y733F)-hGRK1-*Gucy2e*	P14–P25	Cones: up to ~45% of WT	Cone-mediated OKN reflex	Cone preservation	1 year
GC1/GC2DKO mouse	AAV8(Y733F)-hGRK1-*Gucy2e*	P21–P108	Cones: up to ~42% of WT	Cone-mediated OKN reflex	Cone/rod preservation	1 year
			Rods: up to ~44% of WT	Cone and rod-mediated visual acuity		

## WILL *GUCY2D* GENE REPLACEMENT RESTORE RETINAL FUNCTION *IN VIVO*?

The first animal model used to investigate gene replacement for retGC1 was the *retinal degeneration* (*rd*) chicken, also referred to as the GUCY1^*^B chicken. This cone- dominant, avian model carries a null, naturally occurring deletion/insertion mutation in the gene encoding retGC1 (Semple-Rowland et al., 1998). As a consequence of retGC1 deficiency, photoreceptors of post-hatch day 1 (P1) GUCY1^*^B chickens have only 10–20% cGMP relative to that found in age-matched, wild type controls and affected chickens are blind at hatch. They have unrecordable ERGs and lack optokinetic and volitional visual behavior (Ulshafer et al., 1984; Williams et al., 2006). These biochemical and visual disturbances occur prior to photoreceptor loss which doesnot begin until 1 week post-hatch (Cheng et al., 1980; Ulshafer et al., 1984) and proceeds in a central to peripheral fashion with cones completely lost by 3.5 months and rods lost by 8 months (Ulshafer and Allen, 1985a,b).

Due to the technical difficulty associated with performing subretinal injections in chicken, an *in ovo* treatment paradigm was developed. For its ability to stably transduce retinal progenitor cells (Miyoshi et al., 1997), HIV-1-based lentivirus (LV) was used to deliver a cDNA encoding bovine retGC1 under the control of an elongation factor 1 alpha (EF1α) promoter to the neural tube of developing GUCY1^*^B embryos (Williams et al., 2006). Bovine retGC1 (bGC1) was chosen for its verified activity in the presence of chicken GCAPs *in vitro* (Williams et al., 2006). At 1 month post-hatch, ERG testing under both dark and light-adapted conditions revealed that LV-EF1α-bGC1 treatment produced modest increases in photoreceptor-mediated a- wave amplitudes in treated chickens (~6% of wild type). These results were the first demonstration that retGC1 gene replacement could restore retinal function. ERG improvements were associated with a restoration of visually guided behavior, as assessed by optokinetic reflex testing (Wallman and Velez, 1985) and volitional visually guided behavior tests (Williams et al., 2006). In addition, a slowing of retinal degeneration was observed (Williams et al., 2006). Taken together, these results were exciting proof of concept that gene replacement therapy could be effective for treatment of LCA1. However, there were multiple limitations associated with this study that needed to be overcome. First, the therapeutic effect was transient, with ERG and behavioral responses disappearing after the latest time points analyzed (5 weeks post-hatch) and retinal degeneration continuing unabated (Williams et al., 2006; Verrier et al., 2011). Second, multiple facets of the therapeutic strategy lacked clinical translatability- (1) Therapeutic retGC1 was delivered embryonically, a currently untenable task in patients and also requiring in-utero genotyping. (2) An integrating, lentiviral vector was used. While proven effective for transducing retinal precursors, LV has demonstrated little to no ability to transduce post-mitotic photoreceptors (Miyoshi et al., 1997; Bainbridge et al., 2001; Pang et al., 2006; Lipinski et al., 2014). (3) Data was obtained in a non-mammalian model of retGC1 deficiency. Together, this highlighted the need to test therapy using more clinically relevant animal models and vector platform.

## WILL POST-NATALLY DELIVERED retGC1 RESTORE RETINAL FUNCTION IN A MAMMALIAN MODEL OF retGC1 DEFICIENCY?

The first mammalian model retGC1 deficiency to be described was the guanylate cyclase-1 knockout (GC1KO) mouse (Yang et al., 1999). Insertion of a neomycin resistance cassette in exon 5 of *Gucy2e* (the murine homolog of *GUCY2D*) resulted in truncation of retGC1, rendering it null (Yang et al., 1999). Cone-mediated ERGs are unrecordable by 5 weeks but cones are not completely degenerated in this mouse until ~6 months of age (Yang et al., 1999; Coleman et al., 2004). Rods, on the other hand, do not degenerate and maintain variable levels of function (~30–50% of WT; Yang et al., 1999) which is owed to the presence of retGC2 in those cells (Baehr et al., 2007). GCAP1 and GCAP2 transcripts and GCAP1 expression are down-regulated (Coleman et al., 2004) and light-induced translocation of cone arrestin is disrupted as a result of retGC1 deficiency in this model (Coleman and Semple-Rowland, 2005). The temporal dissociation between loss of cone function and structure in GC1KO mice, provides a window of therapy for gene replacement.

For its safety profile and proven ability to transduce post-mitotic photoreceptors following subretinal injection of murine retina (Yang et al., 2002), a serotype 5 adeno associated virus (AAV) was chosen to deliver bovine retGC1 (the same cDNA delivered to the GUCY1^*^B chicken) under the control of either the ubiquitous, small CBA (smCBA) or photoreceptor-specific, murine opsin (MOPS) promoter to the GC1KO mouse (Haire et al., 2006). Post-natal day 21 (P21) treatment with AAV5-bGC1 failed to improve retinal function (ERG) in treated mice. However, AAV-mediated retGC1 expression was restricted to photoreceptor outer segments and restoration of light-induced cone arrestin translocation, a biochemical correlate of functionality in these cells, was observed at 4 weeks post-treatment (Haire et al., 2006), suggesting that the light signaling cascade in GC1KO cones had been at least partially reset following treatment.

Investigators speculated that the species non-specific nature of the delivered transgene may have dampened the therapeutic outcome. Indeed, subretinal delivery of murine *Gucy2e* via an AAV5 vector containing either the ubiquitous smCBA or photoreceptor-specific human rhodopsin kinase (hGRK1) promoter led to robust improvements (~45% of WT) in cone-mediated ERGs (Boye et al., 2010). These improvements were stable over the course of this initial study (at least 3 months post-treatment) and provided the first evidence that an AAV-based vector could restore retinal function to a mammalian model of retGC1 deficiency (Boye et al., 2010). Later studies would go on to show that stable restoration of cone function (ERG) is achievable over the long term (Boye et al., 2011; Mihelec et al., 2011). Subretinally delivered AAV8 containing the hGRK1 promoter and human *GUCY2D* cDNA stably restored cone function (~65% of WT) for at least 6 months (Mihelec et al., 2011). In the longest follow up reported to date, subretinally delivered AAV5 or capsid mutant AAV8(Y733F) containing either the smCBA or hGRK1 promoter and murine *Gucy2e* cDNA stably restored cone function (~45% of WT) for at least 1 year post-treatment (Boye et al., 2011). A notable difference between these two studies was the level of cone function achieved (65 vs. 45% of WT). Mihelec et al. (2011) subretinally delivered AAV-*GUCY2D* to GC1KO mice at P10 (an age prior to natural eye opening) whereas Boye et al. (2011) delivered AAV-*Gucy2e* between P14–P25. Earlier intervention was likely more effective at combating the chronic effects of hyperpolarization that GC1KO cones endure upon light stimulation. Regardless of these differences, the robust and stable functional improvements in cone function following AAV-retGC1 treatment (including with a clinically relevant human cDNA) in GC1KO mice laid the groundwork for development of an AAV-based treatment for LCA1.

As a follow up to these studies, cone-mediated function was also evaluated in the retGC1/retGC2 double knockout (GCDKO mouse following subretinal delivery of AAV8(Y733F)-hGRK1-*Gucy2e* (Boye et al., 2013). Treatment of this model, in which cones and rods are functionally silent and progressively degenerate, also resulted in stable and robust improvements in cone function for at least 1 year post-treatment (Boye et al., 2013). As in all GC1KO studies, AAV-mediated retGC1 expression was restricted to the outer segments of rods and cones in treated GCDKO mice. The GCDKO mouse and gene replacement experiments in this model will be discussed in more detail below.

## DOES RESTORATION OF CONE FUNCTION TRANSLATE INTO USEFUL VISION?

Adeno associated virus-mediated expression of retGC1 in photoreceptors of GC1KO mice effectively reset the phototransduction cascade in cones, allowing them to send electrochemical signals to downstream neurons which were detected via full field ERG electrodes (Boye et al., 2010, 2011; Mihelec et al., 2011). However, the existence of functional neural circuits within the retina does not ensure that electrochemical signals are properly relayed to higher order processing centers in the brain and experienced as “vision.” Even if the biochemical defect within photoreceptors is corrected via restoration of retGC1, it is unclear what role amblyopia (the inability of the retina to send visual information to the brain) will play on the treatment outcome. To gain insight into this question, multiple visually guided behavior tests were used to further characterize therapy in AAV-treated GC1KO mice.

Two tests, each of which evaluates the integrity of different visual processing centers of the brain, were used to determine whether useful cone vision could be restored to treated mice. A virtual optokinetic system was used to evaluate the integrity of subcortical retinal efferents (Douglas et al., 2005). While untreated GC1KO mice lack cone-mediated behavior, significant improvements in cone-mediated spatial frequency thresholds and contrast sensitivities were seen in GC1KO mice treated with AAV5-hGRK1-*Gucy2e*, AAV5-smCBA-*Gucy2e*, AAV8(Y733F)-hGRK1-*Gucy2e* or AAV8-hGRK1-*GUCY2D* (Boye et al., 2010, 2011; Mihelec et al., 2011) with behavior resembling that of normal, sighted congenic mice. Improvements in cone-mediated behavior were observed out to 4 months post-treatment (Mihelec et al., 2011), the latest time point evaluated in this mouse model. Analysis of cone-mediated behavior was also evaluated in GCDKO mice via Morris Water Maze (under photopic conditions), a test used to analyze cortically driven visual behavior (Boye et al., 2013). AAV8(Y733F)-hGRK1-*Gucy2e* treated mice exhibited significantly reduced escape latencies (the amount of time required to escape water bath via a platform demarcated with a flag) relative to untreated controls. Importantly, behavior of treated mice was not significantly different from age-matched WT controls. Notably, these results were obtained in 1 year old GCDKO mice treated 11 months prior, highlighting that retGC1 supplementation confers useful cone-mediated vision to mammalian models of LCA1 over the long term. This is significant as humans rely heavily on their cone-mediated vision for activities of daily living. It is also interesting to note that while cone-mediated ERG improvements in both GC1KO and GCDKO controls ranged between 45 and 65% that of WT controls, cone-mediated behavior was restored to “normal” in AAV-treated mice supporting previous work showing that behavior is a more sensitive indicator of therapy (Williams and Jacobs, 2007). This was also shown to be the case in LV-retGC1 treated GUCY1^*^B chickens whose modest ERG responses corresponded, albeit transiently, to robust visually guided behavior (Williams et al., 2006).

## HOW DO ROD PHOTORECEPTORS RESPOND TO TREATMENT?

Prior to the most comprehensive clinical characterization of LCA1 patients published to date (Jacobson et al., 2013), multiple reports had described that both rod/cone function and rod/cone structure were compromised (Perrault et al., 1999a,b; Milam et al., 2003; Porto et al., 2003; den Hollander et al., 2008; Chung and Traboulsi, 2009; Jacobson et al., 2013). It was clear that, despite the presence of retGC2 in these cells (Lowe et al., 1995), rod photoreceptors of LCA1 patients are negatively impacted by retGC1 deficiency. Thus, the specific effects of post-natal gene replacement therapy on this photoreceptor subclass needed to be analyzed. Prior studies were conducted in a model that lacks rod degeneration and retains variable levels of rod function throughout its life, the GC1KO mouse. For these reasons, it was not possible to address whether gene replacement would prevent degeneration of rods or definitively restore their function, respectively. Significant improvements in rod-mediated ERGs were only obtained when vector was delivered prior to natural eye opening (P10; Mihelec et al., 2011), but it is difficult to ascertain whether these gains are meaningful because GC1KO mice exhibit normal rod-mediated visual behavior. It wasnot until generation of the GCDKO mouse that it became possible to ask these questions (Baehr et al., 2007).

Deletion of both retGC1 and retGC2 in the GCDKO mouse (Baehr et al., 2007) renders both cones and rods functionally silent (ERGs are unrecordable) and degenerative (Baehr et al., 2007). Several cone proteins (GCAP1, GCAP2, cone opsins, cone transducin, cone PDE, GRK1) are downregulated/mislocalized) and PDE6 is absent from rods (Baehr et al., 2007) in this model. By 2 months of age, overall outer segment length is reduced to 30–50% of normal (Baehr et al., 2007). Appreciable thinning of the outer nuclear layer (ONL) is apparent by ~3.5 months of age and by 6 months, only 3–4 photoreceptor nuclei remain (Boye et al., 2013).

For its success in the studies described above, AAV8(Y733)-hGRK1 was chosen to subretinally deliver *Gucy2e* to cohorts of GCDKO mice of various age groups (P18, P21–P25, P37–P49, and P108). ERG revealed that, like the cone results described above, rod function was also stably restored to ~40% of normal (at least 1 year post-treatment; Boye et al., 2013). Morris Water Maze testing revealed that these functional gains translated to useful rod-mediated vision, with AAV-retGC1- treated GCDKO mice performing the task at speeds that did not differ significantly from age-matched WT controls (Boye et al., 2013). As in the treated GC1KO mice, WT-like behavior was achievable in GCDKO mice that exhibited only partial ERG recovery. Optical coherence tomography (OCT) was used to monitor the rate of photoreceptor degeneration (predominantly rods) in treated vs. untreated mice. By 7–12 months post-treatment, there was significant structural preservation in all treated GCDKOs, barring those treated at the latest time point (P108), although retinas in these mice still exhibited greater preservation than controls (Boye et al., 2013). Importantly, the rate of photoreceptor cell loss in GCDKO mice treated as late as P49 was no different from that of age-matched WT controls. GCAP1 and GCAP2 were expressed at WT-like levels at 1 year post-injection and cone opsins were present only in treated cones (Boye et al., 2013). Taken together, this study showed for the first time that, along with the rescue effects seen on cones, AAV-mediated retGC1 expression can restore rod photoreceptor function and rod-mediated visual behavior and preserve rod structure over the long term in an animal model of LCA1.

## WHAT IS THE FUNCTIONAL EFFICIENCY OF AAV- DELIVERED retGC1 ENZYME?

In considering clinical application of a gene therapy, an important question becomes “how efficient is the vector-derived, recombinant gene product relative to the native protein?” The ability to assess this *in vivo* provides a powerful bioassay which can be used to establish dose-response relationships and equivalencies for batches of clinical vectors. Guanylate cyclase activity assays are performed on dark-adapted retinas to determine how much cGMP is produced by retGCs in the presence of a [α-^32^P]GTP substrate (Olshevskaya et al., 2004; Peshenko et al., 2011). This assay doesnot discriminate between the activity of retGC1 and retGC2. Therefore, isolating the functional efficiency of exogenous retGC1 requires delivery to photoreceptors that lack both endogenous retGC1 and retGC2, such as those in the GCDKO mouse. Boye et al. (2013) compared retGC activity in retinas of GCDKO mice that were treated subretinally between P30–P60 with AAV8(Y733F)-hGRK1-*Gucy2e*, relative to untreated GCDKO or WT controls and those that received a serotype-matched control vector expressing GFP. Maximal retGC activity in AAV-*Gucy2e* treated retinas was ~63% of normal. The known contribution to total cyclase activity by retGC2 in this assay is between 20 and 28% (Peshenko et al., 2011). In addition, the area of retGC1 expression in treated retinas is known to be restricted to the subretinal injection bleb (Timmers et al., 2001; Cideciyan et al., 2008). “Good” subretinal injections typically result in ~80–90% detachment meaning not all photoreceptors expressed the enzyme. Taking these two factors into account, the level of retGC1 activity suggested its near-complete restoration in the area exposed to vector. When the maximal activity of AAV-retGC1 was normalized to that seen in WT retinas, it was determined that the calcium sensitivity of the exogenous and endogenous enzymes were identical (Boye et al., 2013). Moving forward, this assay may be used to evaluate the relative potency of clinical vectors.

## WHAT ARE THE CONSEQUENCES OF retGC1 DEFICIENCY ON HUMAN ROD AND CONE PHOTORECEPTOR STRUCTURE AND FUNCTION?

While there is general consensus that LCA1 is associated with severely attenuated or ablated ERG (Perrault et al., 1999b, 2000; Hanein et al., 2004; Yzer et al., 2006; den Hollander et al., 2008), reports on the extent of photoreceptor degeneration associated with this form of LCA have been conflicting (Milam et al., 2003; Porto et al., 2003; Simonelli et al., 2007; Pasadhika et al., 2010). The most thorough clinical characterization of LCA1 performed to date focused on a cohort of patients ranging in age from 6 months to 37 years with different mutations in *GUCY2D* and has provided new insight on the effects of retGC1 deficiency on retinal structure and function (Jacobson et al., 2013).

Similar to previous reports Simonelli et al. (2007), Pasadhika et al. (2010), Jacobson et al. (2013) found that LCA1 patients retained normal retinal laminar architecture aside from foveal cone outer segment abnormalities and, in some cases, foveal cone loss. Within rod-dominant retina, ONL thickness and outer segment lengths were normal in all patients (Jacobson et al., 2013). Unlike previous reports, Jacobson et al. (2013) found that LCA1 patients can retain substantial rod function. Full field sensitivity testing (FST) revealed all patients effectively used their rods to detect blue stimuli, albeit at reduced sensitivities. These psychophysical findings were supported by ERG, pupillometry and mobility testing, all of which revealed variable levels of retained rod function that did not correlate with the patient’s age (Jacobson et al., 2013). In contrast, the majority of patients lacked cone sensitivity (FST) which correlated to severely reduced visual acuity and a lack of color perception. FST, microperimetry and mobility tests revealed a small subset had detectable, but reduced cone function with central fixation and some color perception. None of the patients had recordable cone-mediated ERGs (Jacobson et al., 2013).

To better understand these differential cone phenotypes, Jacobson et al. (2013) investigated the activity of patient-specific retGC1 mutants *in vitro*. Their activity was assayed by transfecting cDNA for each form, along with GCAPs in HEK293 cells under appropriate ionic conditions and quantifying the amount of cGMP produced relative to that achieved with wild type retGC1. Of the patient-specific mutants tested, some exhibited reduced catalytic activity (4–5 fold lower than WT) while others demonstrated no loss of function (Jacobson et al., 2013). Not surprisingly, those mutants that retained biochemical activity were expressed by LCA1 patients with measurable cone function. It is expected that those with normal activity *in vitro* may exhibit defective folding, expression or impaired trafficking to photoreceptor outer segments *in situ*. Investigations are currently underway to understand the fate of these mutants *in vivo* via AAV-mediated expression in GCDKO mice. retGC1 mutants that exhibited no activity *in vitro* corresponded to patients with profound loss of cone function (Jacobson et al., 2013).

## WHAT FORM MIGHT A CLINICAL TRIAL FOR *GUCY2D*-LCA1 TAKE?

A few important choices must be made prior to clinical application of a gene replacement therapy for LCA1. These include the injection route and location, AAV serotype and promoter, and therapeutic endpoints. Route and location of injection should be dictated by the calculated risk(s)/benefit. The structural integrity of the patient’s retina and locus of existing vision must be considered. Evidence suggests *GUCY2D*-LCA1 is unlike any form of LCA studied in detail to date (Jacobson et al., 1998, 2003, 2005, 2007a,b, 2009b, 2011; Cideciyan et al., 2007). Their hallmark retinal preservation suggests that LCA1 patients may be good candidates for subretinal injection of AAV-*GUCY2D* (Simonelli et al., 2007; Pasadhika et al., 2010; Jacobson et al., 2013). Because retGC1 deficiency leads to profound visual impairment, stemming primarily from cones, the central retina should be the treatment target. In the small subset of patients that exhibit foveal cone losses, para- or peri-foveal areas may be targeted in the hopes that, like some *RPE65*-LCA2 patients, they will develop an eccentric locus of fixation (Cideciyan et al., 2009).

AAV5 and AAV8-based vectors were used in the aforementioned studies for developing a treatment for LCA1. These vectors have proven utility for photoreceptor-targeted therapy following subretinal injection in multiple mouse and dog models of inherited retinal disease (Min et al., 2005; Alexander et al., 2007; Boye et al., 2010, 2013; Gorbatyuk et al., 2010; Komaromy et al., 2010; Mao et al., 2011; Pang et al., 2011, 2012; Yao et al., 2011; Beltran et al., 2012; Petit et al., 2012; Lheriteau et al., 2014). Because AAV transduction profiles and the activity of promoters can vary across species, the final decision should be dictated by the serotype and promoter combination’s behavior in a species most closely related to man, non-human primate (NHP). In LCA1, photoreceptors of the central retina are the treatment target. Therefore, serotype and promoter combinations that effectively transduce cone photoreceptors in NHP are required. Transduction of both AAV5 and AAV8 vectors have recently been described in subretinally injected NHP (Boye et al., 2012; Vandenberghe et al., 2013). Only partial cone transduction was achieved following subretinal delivery of elevated doses of AAV8 containing the ubiquitous CMV promoter driving GFP (10^11^ vg delivered; Vandenberghe et al., 2011, 2013). At a lower dose (10^10^ vg delivered), AAV8-CMV-GFP failed to transduce foveal, parafoveal or perifoveal cones (Vandenberghe et al., 2011). In contrast, foveal, parafoveal, perifoveal and peripheral cones of NHP (as well as rods) were all effectively transduced following subretinal delivery of an AAV5 vector containing the photoreceptor-specific hGRK1 promoter (10^10^ vg delivered; Boye et al., 2012). This mirrors earlier findings in rodent that hGRK1 has exclusive activity in cones and rods (Khani et al., 2007; Tan et al., 2009; Boye et al., 2010, 2011; Pawlyk et al., 2010; Sun et al., 2010). Taken together, these results support the use of subretinally delivered AAV5-hGRK1*-GUCY2D* in a clinical setting.

Because LCA1 patients exhibit profound cone-mediated visual impairment and variable levels of rod-mediated vision, typical measures of vision must be supplemented with assays that are more suited to this severe disease. Most patients have nystagmus, making full field ERG recordings difficult. Additionally, because many LCA1 patients have unrecordable ERGs to begin with, it would be impossible to measure any negative treatment-associated changes with this assay. Tests which control for nystagmus such as microperimetry can be used to assess cone-mediated central vision (Cideciyan et al., 2012). Tests such as chromatic FST which was used in the most recent clinical characterization (Jacobson et al., 2013) and in the clinical trial for RPE65-LCA2 (Jacobson et al., 2012) could be used to measure localized improvements in rod function. The limits of variability for this assay have already been defined in patients with various retinal degenerations, including LCA (Roman et al., 2005; Roman et al., 2007; Jacobson et al., 2009a). OCT should be performed to monitor the safety of injections performed under the fovea or para/peri- fovea, with emphasis placed on the former given the slow return of cone outer segment structure observed in LCA2 patients treated subfoveally (Jacobson et al., 2012). The potential effects of amblyopia must also be considered. It is possible that AAV-mediated expression of wild type retGC1 in cones of LCA1 patients will reset their signaling cascade allowing for electrochemical signals to propagate through the retina, but that these signals will not be effectively relayed to the brain. Magnetic resonance imaging studies could be used to confirm the integrity of higher order visual processing pathways prior to treatment. Fully understanding how the brain of an LCA1 patient will accommodate *GUCY2D* gene replacement and whether this will differ depending the patient’s mutation and/or level of cone preservation will only be accomplished through a clinical trial.

## CONCLUSION

Information gleaned from proof of concept experiments in multiple animal models of retGC1 deficiency have laid the groundwork for development of an AAV-based treatment for *GUCY2D*-LCA1 (Haire et al., 2006; Williams et al., 2006; Boye et al., 2010, 2011; Mihelec et al., 2011). Thorough clinical characterization of patients has provided new insights into the pathophysiology of this severe early onset inherited retinal disease, pointing to the central retina as the target for treatment and highlighting the importance of selecting appropriate outcome measures by which to score therapeutic efficacy in a clinical trial (Jacobson et al., 2013). Studies in NHP have revealed an optimal AAV serotype (AAV5) and promoter (hGRK1) combination for delivery of therapeutic transgene to all cone subclasses and rods (Boye et al., 2012). The majority of genes (13/19) known to account for some form of LCA, including *GUCY2D*, encode photoreceptor specific proteins (den Hollander et al., 2008; Wang et al., 2009; Estrada-Cuzcano et al., 2011; Sergouniotis et al., 2011; Abu-Safieh et al., 2013). In fact, inherited retinal diseases as a whole are caused primarily by defects in proteins expressed by photoreceptors (Wright et al., 2010), a cell type yet to be targeted by AAV in a clinical setting. Thus, developing a treatment for *GUCY2D*-LCA1 will serve as a framework for other photoreceptor-targeted gene replacement strategies.

## Conflict of Interest Statement

Shannon E. Boye is a co-inventor on US patent # 61/327,521 which covers some aspects of the material discussed within.
